# An Integrated Fibrosis Signature for Predicting Survival and Immunotherapy Efficacy of Patients With Hepatocellular Carcinoma

**DOI:** 10.3389/fmolb.2021.766609

**Published:** 2021-12-14

**Authors:** Long Liu, Zaoqu Liu, Lingfang Meng, Lifeng Li, Jie Gao, Shizhe Yu, Bowen Hu, Han Yang, Wenzhi Guo, Shuijun Zhang

**Affiliations:** ^1^ Department of Hepatobiliary and Pancreatic Surgery, The First Affiliated Hospital of Zhengzhou University, Zhengzhou, China; ^2^ Henan Research Centre for Organ Transplantation, Zhengzhou, China; ^3^ Henan Key Laboratory of Digestive Organ Transplantation, Zhengzhou, China; ^4^ Department of Interventional Radiology, The First Affiliated Hospital of Zhengzhou University, Zhengzhou, China; ^5^ Department of Infection Management, The Second Affiliated Hospital of Zhengzhou University, Zhengzhou, China; ^6^ Internet Medical and System Applications of National Engineering Laboratory, Zhengzhou, China

**Keywords:** hepatocellular carcinoma, fibrosis, immunotherapy, prognosis, immune

## Abstract

**Introduction:** Fibrosis, a primary cause of hepatocellular carcinoma (HCC), is intimately associated with inflammation, the tumor microenvironment (TME), and multiple carcinogenic pathways. Currently, due to widespread inter- and intra-tumoral heterogeneity of HCC, the efficacy of immunotherapy is limited. Seeking a stable and novel tool to predict prognosis and immunotherapy response is imperative.

**Methods:** Using stepwise Cox regression, least absolute shrinkage and selection operator (LASSO), and random survival forest algorithms, the fibrosis-associated signature (FAIS) was developed and further validated. Subsequently, comprehensive exploration was conducted to identify distinct genomic alterations, clinical features, biological functions, and immune landscapes of HCC patients.

**Results:** The FAIS was an independent prognostic predictor of overall survival and recurrence-free survival in HCC. In parallel, the FAIS exhibited stable and accurate performance at predicting prognosis based on the evaluation of Kaplan–Meier survival curves, receiver operator characteristic curves, decision curve analysis, and Harrell’s C-index. Further investigation elucidated that the high-risk group presented an inferior prognosis with advanced clinical traits and a high mutation frequency of *TP53*, whereas the low-risk group was characterized by superior CD8^+^ T cell infiltration, a higher TIS score, and a lower TIDE score. Additionally, patients in the low-risk group might yield more benefits from immunotherapy.

**Conclusion:** The FAIS was an excellent scoring system that could stratify HCC patients and might serve as a promising tool to guide surveillance, improve prognosis, and facilitate clinical management.

## Introduction

Worldwide, liver cancers are the sixth most prevalent cancer and rank second in cancer-related mortality (Sung et al., 2021). Hepatocellular carcinoma (HCC) is the major histologic category of primary liver cancers, which mainly arise from chronic liver diseases, mostly as a result of HBV/HCV infection, alcoholic liver disease, and liver cirrhosis (Villanueva, 2019). Although the diagnosis and treatment of HCCs are increasingly diversified, the clinical benefits are limited, and the 5-year relapse rate exceeds 70% (Brown et al., 2019). In clinical practice, the Barcelona Clinic Liver Cancer (BCLC) staging system is a basic measurement to evaluate risk and manage the prognosis of HCC patients (Villanueva, 2019). However, the current staging system is restricted to stratifying patients and may hinder optimal clinical decisions as HCC patients present significant heterogeneity (Craig et al., 2020). The clinical heterogeneity of HCC is actually reflected from molecular heterogeneity. Therefore, searching for a new tool *via* multiple gene panels to stratify patients and further improve clinical management is imperative.

Fibrosis is a highly dynamic process and is associated with spatiotemporal regulation, the involvement of multiple carcinogenic signaling pathways, and the interaction of inflammation (Koyama and Brenner, 2017; Henderson et al., 2020). For instance, proteins involved in the mTOR signaling pathway, mTOR complex 1 (mTORC1) and mTOR complex 2 (mTORC2), have pronounced impacts on fibrogenesis (Huang et al., 2020). The mTORC1/4E-BP1 axis is a universal molecular pathway and is involved in the fibrosis progression of different tissues (Woodcock et al., 2019). Moreover, liver fibrosis could progress to cirrhosis and is a primary cause of HCC, playing an important role in the premalignant environment (Affo et al., 2017). Previous studies demonstrated that both the essential mediator of fibrogenesis transforming growth factor-*β* (TGF-*β*) and the aberrant glycolysis metabolism could activate hepatic stellate cells (HSCs), aggravating liver fibrosis (Koyama and Brenner, 2017; Chang and Yang, 2019). Various cells, cytokines, and the extracellular matrix are significant and consist of a fibrotic microenvironment, leading to the development of HCC (Filliol and Schwabe, 2019). Cancer-associated fibroblasts (CAFs) in HCC facilitate tumor metastasis *via* hedgehog and TGF-*β* pathways (Liu et al., 2016b). Inflammation is also involved in the process of fibrogenesis and regulates immune cells in the tumor microenvironment (TME) (Mack, 2018). Different phenotypic and functional macrophages are key players in fibrogenesis, such as pro-inflammatory M1 macrophages and anti-inflammatory M2 macrophages, which accelerate the occurrence and development of fibrosis (Wynn and Vannella, 2016; Schuppan et al., 2018). Exploring the biological characteristics and immune landscape of each patient might help increase the understanding of fibrogenesis.

HCC has a high relapse rate and adverse long-term prognosis, with a 5-year survival of approximately 18% (Jemal et al., 2017). New therapeutic strategies are desperately needed to improve overall survival (OS). Personalized immunotherapy is currently an encouraging and revolutionized treatment for solid tumors (Chai et al., 2020). Nevertheless, the therapeutic efficacy is disappointing as only a small proportion of individuals yield prominent benefits (Finn et al., 2020). The inter- and intra-tumoral heterogeneity of HCC may be responsible for different treatment effects (Craig et al., 2020). Many immune cells are critical components of the TME and display tight interactions with tumor cells, which are likely to involve the development of tumor heterogeneity (Hinshaw and Shevde, 2019; Liu et al., 2021d). Previous studies have suggested that TME markers exhibit striking advantages in assessing immunotherapy in HCC patients (Zhang et al., 2019). As a common approach of immunotherapy, immune checkpoint inhibitor (ICI) treatment has made encouraging progress, which aims to help immune surveillance and target specific immune checkpoints, such as programmed death-ligand 1 (*PD-L1*) and cytotoxic T-lymphocyte-associated protein 4 (*CTLA-4*) (Ribas and Wolchok, 2018). With the deep investigation of the TME, patients with objective and durable immunotherapy responses may be identified, which is conducive to improving clinical management.

Advances in bioinformatics and machine learning have made it possible to explore tumor heterogeneity at the genomic level and identify a robust multigene signature for HCC patients. In this context, we performed three machine learning algorithms, including stepwise Cox regression, LASSO, and random survival forest (RSF) algorithms, to construct and validate a stable signature. Based on the expression of fibrosis-associated genes (FAGs), an individualized fibrosis-associated signature (FAIS) for HCC patients was identified. Furthermore, the clinical and molecular characteristics, underlying biological functions, immune cell infiltration, and immune checkpoint profiles of FAIS were explored. Using the T-cell inflammatory signature (TIS) and Tumor Immune Dysfunction and Exclusion (TIDE) algorithms, the immunotherapy efficacy was evaluated for each patient. The initial establishment of the FAIS for stratifying risk could facilitate clinical benefits, help improve therapeutic strategies, and promote prognostic management of HCC patients. Collectively, this study might support optimized decision making in immunotherapy and further facilitate better clinical outcomes for HCC patients.

## Materials and Methods

### Public Data Collection and Processing

In this study, gene expression data were retrieved from TCGA (https://portal.gdc.cancer.gov/) and GEO (https://www.ncbi.nlm.nih.gov/geo/). All samples enrolled were available for mRNA sequencing. In the TCGA-LIHC cohort, the corresponding clinical information, copy number alteration (CNA), and somatic mutation were obtained from the online portal cBioPortal (http://www.cbioportal.org/), which contained 363 HCC patients with survival information. The GSE14520 cohort integrated 221 HCC patients who possessed comprehensive clinical information, including overall survival (OS) and recurrence-free survival (RFS). All the gene expression data are from the patients at diagnosis. The detailed baseline data from TCGA-LIHC and GSE14520 are summarized in Supplementary Table S1. OS and RFS were regarded as primary outcome events for prognostic factor analysis. OS was measured from the time of surgery to death, and RFS was estimated from the time of surgery to cancer recurrence.

### Construction and Evaluation of Signatures

To obtain a stable and valuable signature, we used three machine learning algorithms for analysis, including stepwise Cox regression, LASSO, and RSF algorithms. These algorithms were fitted based on 10-fold cross-validations. First, some fibrosis-associated genes (FAGs) were retrieved from the Molecular Signatures Database (MSigDB, http://www.gsea-msigdb.org/gsea/msigdb/search.jsp). The other FAGs were extracted from the study of Job-S et al. (Job et al., 2020). Second, the limma package was used for differential expression analysis to identify candidate FAGs. Then, the overlapping differentially expressed genes (DEGs) were screened between the TCGA-LIHC and GSSE14520 cohorts. Kaplan–Meier analysis and univariate Cox regression were applied to further assess the prognostic value of these genes. Finally, we constructed three signatures, which were evaluated by the Kaplan–Meier survival curve, receiver operator characteristic (ROC) curve, decision curve analysis (DCA), and Harrell’s C-index. The Kaplan–Meier survival curve was utilized to display the predictive ability by the Survival R package. Using the time ROC package, time-dependent ROC curves for survival variables were created to assess the performance of signatures based on the area under the ROC curves (AUCs). DCA was employed to evaluate the clinical utility of the three signatures by the ggDCA R package. The Harrell’s C-index of three signatures was calculated and compared to reflect their accuracy and stability by the compareC R package.

### Clinical and Molecule Characteristics

According to the median of the signature’s risk score, the HCC patients were assigned into high-risk and low-risk groups. The heatmap with multiple clinical traits was generated by the Pheatmap R package. The survival status, age, gender, BMI, AJCC stage, histology grade, and vascular invasion were displayed as patient annotations. Univariate and multivariate Cox regression analyses were used to identify the independent prognostic factors for OS and RFS. For genomic data, the maftools R package was utilized to explore the landscape of somatic mutations (Mayakonda et al., 2018). Additionally, the CNA status was compared between the high-risk and low-risk groups with chi-square tests.

### Biological Function Enrichment Analysis

To further decipher potential biological functions between the high-risk and low-risk groups, Gene Ontology (GO) and Kyoto Encyclopedia of Genes and Genomes (KEGG) enrichment analyses were performed with the clusterProfiler R package. The normalized enrichment score (NES) was calculated according to 1,000 permutations. A false discovery rate (FDR) < 0.05 was defined as statistically significant. In addition, the DEGs of the two groups were ranked in accordance with the log2-foldchange (logFC) value, and the gene set enrichment analysis (GSEA) algorithm was applied to decode the potential molecular mechanisms. The hallmark pathway gene sets were obtained from MSigDB and submitted to identify dramatically carcinogenic pathways using the GSVA algorithm implemented in the GSVA R package.

### Evaluation of Immune Cell Infiltration and Immune Checkpoint Expression

According to the research by Charoentong et al.(Charoentong et al., 2017), immune gene signatures were collected from 28 immune cell subgroups, including innate immune cells and adaptive immune cells (Supplementary Table S2). The gene expression profiles and immune gene signatures were utilized to evaluate the infiltration abundance of 28 immune cells. Using a single sample gene set enrichment analysis (ssGSEA) algorithm (Zuo et al., 2020), the scores were calculated and applied to quantify the infiltration abundance of immune cells in the TME. To elucidate immunotherapy implications and facilitate clinical applications, we retrieved 27 co-stimulatory and 14 co-inhibitory immune checkpoints from the literature Xiao Y et al (Xiao et al., 2019). Based on expression profiles, the heatmaps and boxplots were generated with the ggplot2 R package. The correlations between the risk score and immune infiltration abundance and immune checkpoint expression were assessed by Spearman’s correlation analysis.

### Estimation of Immunotherapy Efficacy

To evaluate the latent response to ICI treatment for HCC patients, two approaches were conducted, encompassing the T-cell inflammatory signature (TIS) and Tumor Immune Dysfunction and Exclusion (TIDE) analysis. Ayers et al. first proposed TIS and further predicted clinical response to PD-1 blockade (Ayers et al., 2017). The signature consists of 18 inflammatory genes conspicuously associated with antigen presentation, chemokine expression, cytotoxic activity, and adaptive immune resistance. The TIDE (http://tide.dfci.harvard.edu/) algorithm is used to model tumor immune evasion, which gathers two primary mechanisms of immune evasions: T cell dysfunction and T cell exclusion. TIDE is a prevalent tool that has been applied in mass studies to evaluate immunotherapy efficacy (Liu et al., 2021a; Liu et al., 2021b; Liu et al., 2021c). Patients with higher TIDE scores portended stronger potential for tumor immune evasion and worse immunotherapy response (Jiang et al., 2018).

### Validation of the FAIS *via* the In-House Cohort

To further verify the robustness of FAIS, quantitative real-time PCR was performed to quantify the expression level of 11 genes. A total of 58 HCC tissues were obtained from patients who underwent surgical treatment at The First Affiliated Hospital of Zhengzhou University, and all the patients had written informed consent. None of the patients received any preoperative chemotherapy or radiotherapy. The detailed baseline is shown in Supplementary Table S1. All the tissue samples were immediately obtained and frozen in liquid nitrogen and subsequently stored at −80°C. This project was approved by the Ethics Committee Board of The First Affiliated Hospital of Zhengzhou University. RNA extraction and reverse transcription were conducted based on the manufacturer’s protocol. The sequences of quantitative real-time PCR primers are summarized in Supplementary Table S3. See Supplementary Material for details.

### Statistical Analysis

The Survival, glmnet, and randomForestSRC R packages were used to perform stepwise Cox regression, LASSO, and RSF, respectively. The Kaplan–Meier method and log-rank test were applied to evaluate the different OS and RFS rates between the two groups. The Wilcoxon rank-sum test was adopted to estimate the difference of continuous variables when comparing two groups. For comparison of more than two groups, the Kruskal–Wallis test was conducted. Fisher’s exact test or Pearson’s chi-squared test was carried out to compare categorical variables. All statistical tests were two-sided tests, and *p* < 0.05 was considered as statistically significant. All data cleaning, statistical analyses, and plots were handled in R 4.0.5 software.

## Results

### Multiple Machine Learning Algorithms Construct Robust Signatures

**FIGURE 1:**
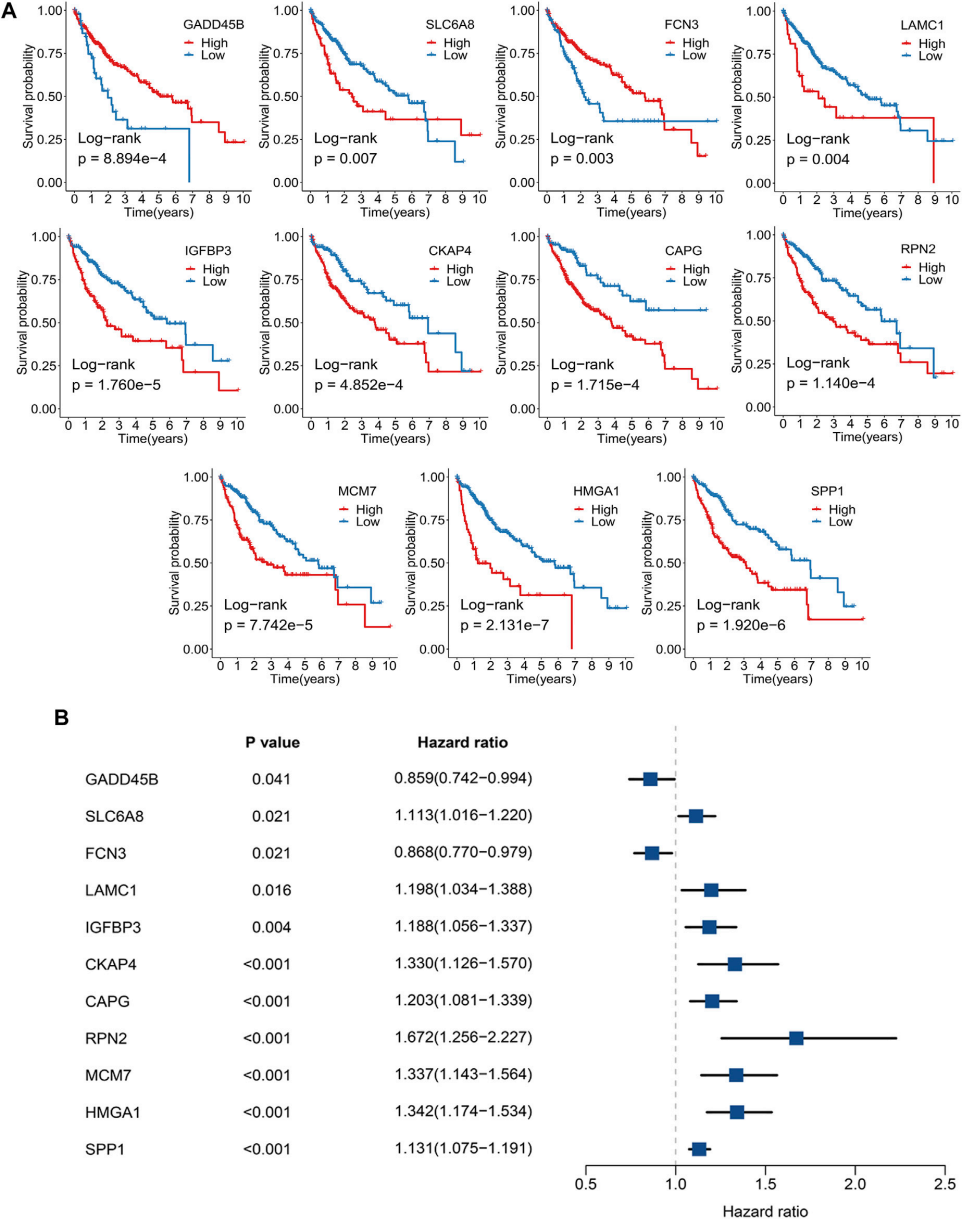
Identification of FAGs with a significantly prognostic value. **(A)** Kaplan–Meier curves of OS according to the 11 FAGs in TCGA-LIHC. **(B)** Univariate Cox regression of 11 FAGs regarding OS in TCGA-LIHC.

**FIGURE 2:**
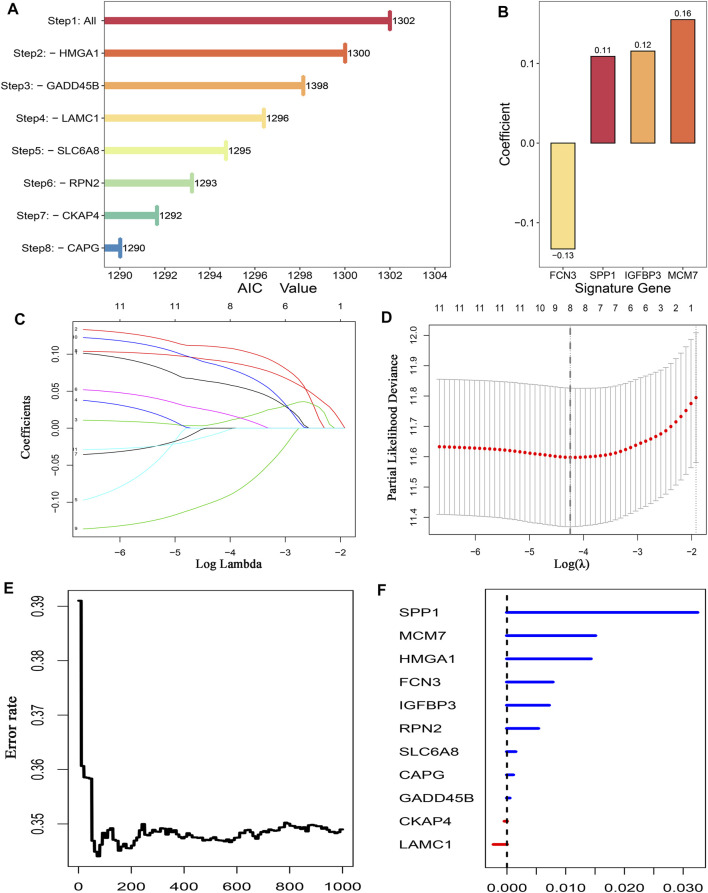
Establishment of prognostic signatures by three machine learning algorithms. **(A)** AIC of stepwise Cox regression analyses. **(B)** Coefficient of four genes finally obtained in stepwise Cox regression analyses. **(C)** LASSO coefficient profiles of the candidate genes for the construction of prognostic signature. **(D)** Determination of the optimal lambda is obtained when the partial likelihood deviance reached the minimum value and further generated the key genes with nonzero coefficients. The dotted vertical line is drawn at the optimal lambda value. **(E)** Relationship between the error rate and the number of classification trees. **(F)** Relative importance values of 11 out-of-bag genes.

A total of 351 FAGs were retrieved in this study. Using the limma R package, there were 95 and 52 DEGs between normal and tumor samples in TCGA-LIHC and GSE14520, respectively. The overlapping DEGs were further screened as candidate genes, including 15 downregulated genes and 20 upregulated genes. To further explore the underlying prognostic value of these 35 FAGs, Kaplan–Meier analysis was carried out, and the results exhibited that 11 FAGs had a potential prognostic value (*p* < 0.05) (Figure 1A). In addition, univariate Cox regression revealed that *FCN3* and *GADD45B* were protective prognostic indicators, while the remaining genes were all risk factors (Figure 1B). Based on the expression of these genes in TCGA-LIHC, we developed signatures for predicting prognosis by three machine learning algorithms. Stepwise Cox regression based on the Akaike information criterion (AIC) showed that signature-1 containing four FAGs was optimal, including *FCN3*, *SPP1*, *IGFBP3*, *and MCM7* (Figure 2A). The coefficients of signature-1 were -0.13, 0.11, 0.12, and 0.16, respectively (Figure 2B). Another algorithm LASSO identified that signature-2 contained eight FAGs, encompassing *RPN2*, *MCM7*, *HMGA1*, *CAPG*, *SPP1*, *FCN3*, *IGFBP3*, and *GADD45B* (Figure 2C). The optimal lambda was obtained when the partial likelihood deviance reached the minimum value. The results suggested that the optimal lambda value was 0.014, and then signature-2 was constructed (Figure 2D). Using RSF, signature-3 contained 11 FAGs, and the error rate is exhibited in Figure 2E. The importance of each variable was estimated and is shown in Figure 2F. Taken together, we obtained three signatures to predict the OS of each HCC patient in the TCGA-LIHC cohort.

### The Comparison and Validation of Multiple Signatures

**FIGURE 3:**
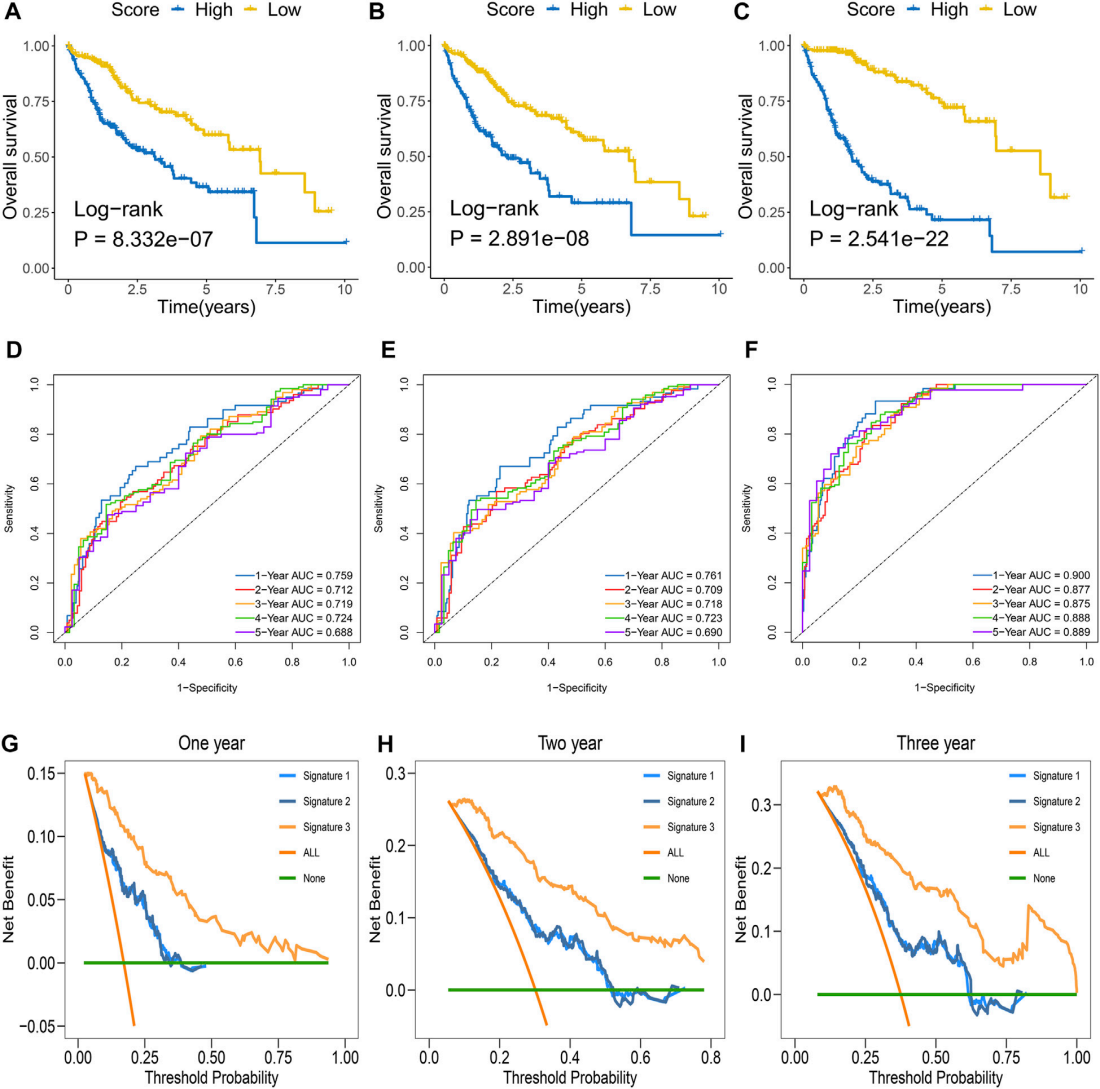
Evaluation and comparison of signatures in the TCGA-LIHC cohort. **(A–C)** Kaplan–Meier curves of OS according to signature-1 **(A)**, signature-2 **(B)**, and signature-3 **(C)**. **(D–F)** Time-dependent ROC analysis for predicting OS at 1–5 years according to signature-1 **(D)**, signature-2 **(E)**, and signature-3 **(F)**. **(G–I)** DCA curves of signatures for evaluating 1- **(G)**, 2- **(H)**, and 3-year **(I)** OS.

For OS as an endpoint event, Kaplan–Meier analysis suggested that the three signatures presented a significant prognostic value in the TCGA-LIHC cohort (Figures 3A–C). Similar results were displayed in the GSE14520 cohort (Supplementary Figures S2A–C). We assessed the three signatures across multiple dimensions and screened the optimal signature. The discrimination was evaluated by ROC and C-index, and clinical utility was assessed by DCA. In the TCGA-LIHC cohort, the ROC curves of the three signatures exhibited that the AUCs for predicting OS at 1–5 years were 0.759, 0.712, 0.719, 0.724, and 0.688 in signature-1; 0.761, 0.709, 0.718, 0.723, and 0.690 in signature-2; and 0.900, 0.877, 0.875, 0.888, and 0.889 in signature-3, respectively (Figures 3D–F). Moreover, the 95% confidence interval (CI) of the C-index and clinical utility value were calculated and compared. The C-index values were 0.699 (95% CI: 0.674–0.724), 0.701 (95% CI: 0.676–0.726), and 0.839 (95% CI: 0.824–0.854) among the three signatures, respectively (Supplementary Figure S1A). As instructed, signature-3 performed superior accuracy and clinical benefits compared with the other signatures (Figures 3G–I and Supplementary Figure S1A). In the GSE14520 cohort, the ROC curves of the three signatures exhibited that the AUCs for predicting OS at 1–5 years were 0.672, 0.720, 0.708, 0.699, and 0.686 in signature-1; 0.673, 0.722, 0.704, 0.692, and 0.681 in signature-2; and 0.699, 0.718, 0.700, 0.655, and 0.652 in signature-3, respectively (Supplementary Figures S2D–F). The C-index values were 0.664 (95% CI: 0.633–0.695), 0.660 (95% CI: 0.629–0.691), and 0.643 (95% CI: 0.611–0.675) among the three signatures, respectively (Supplementary Figure S1B). The three signatures had similar performance in predicting OS (Supplementary Figures S1B, S2G–I). After rigorous comparison and validation, signature-3 presented the highest predictive accuracy and the best clinical utility and was defined as the fibrosis-associated signature (FAIS) for subsequent analysis.

### The Clinical Characteristics and Implications

**FIGURE 4:**
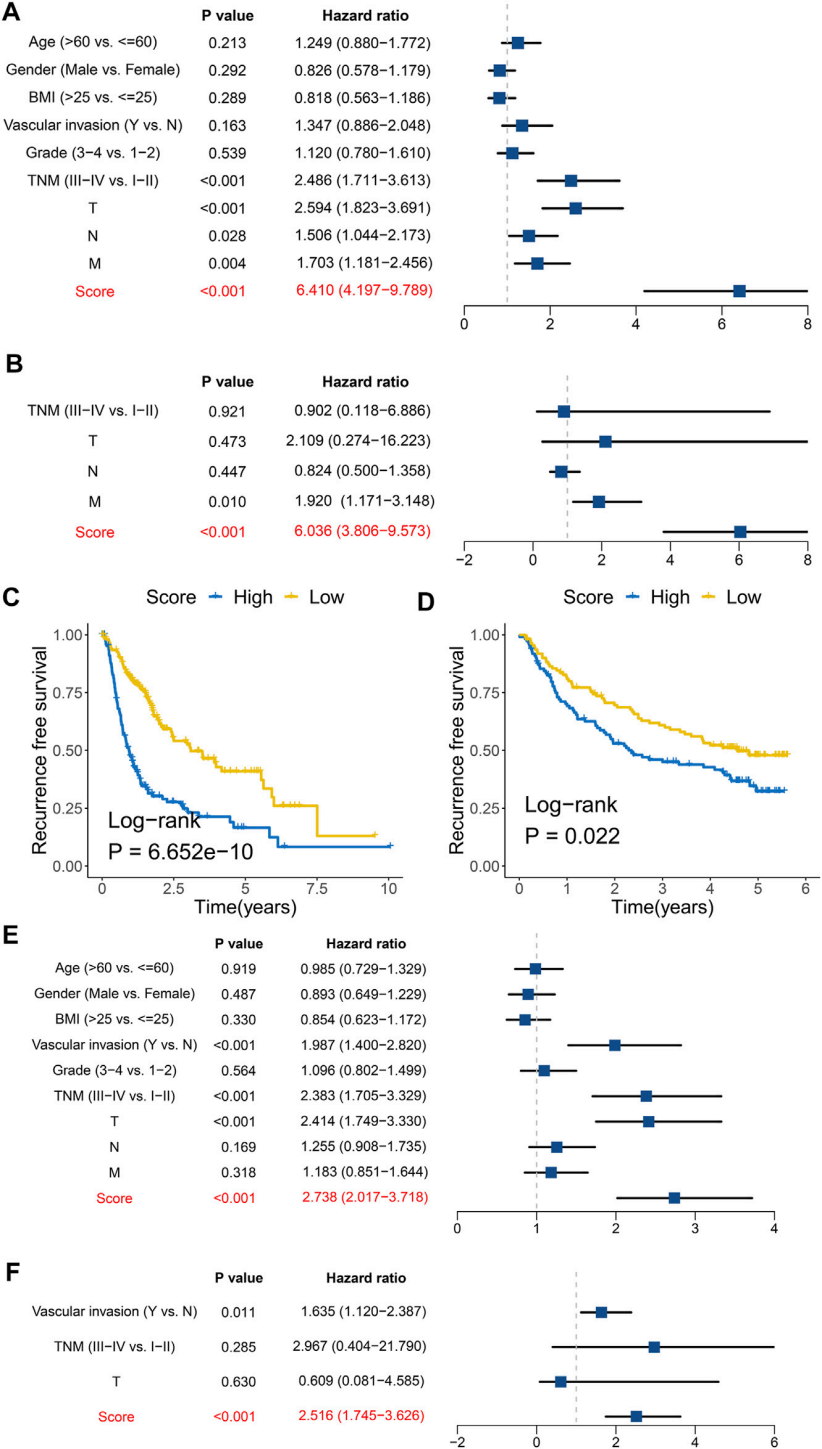
Prognosis significance of overall survival and recurrence-free survival for the FAIS. **(A)** Univariate Cox regression analyses of OS in the TCGA-LIHC cohort. **(B)** Multivariate Cox regression analyses of OS in the TCGA-LIHC cohort. **(C)** Kaplan–Meier curve of RFS according to the FAIS in the TCGA-LIHC cohort. **(D)** Kaplan–Meier curve of RFS according to the FAIS in the GSE14520 cohort. **(E)** Univariate Cox regression analyses of RFS in the TCGA-LIHC cohort. **(F)** Multivariate Cox regression analyses of RFS in the TCGA-LIHC cohort.

To further characterize and reveal the clinical significance of the FAIS, we combined clinical factors for assessing the performance at predicting OS and RFS. Univariate and multivariate Cox regression analyses suggested that the FAIS was an independent predictive factor for OS (Figures 4A,B). The Kaplan–Meier analysis of RFS indicated that the low-risk group had a favorable prognosis (Figures 4C,D). All results demonstrated that the FAIS was also an independent predictive factor for RFS (Figures 4E,F). The above results elucidated that FAIS may be an excellent clinical management and translation tool.

**FIGURE 5:**
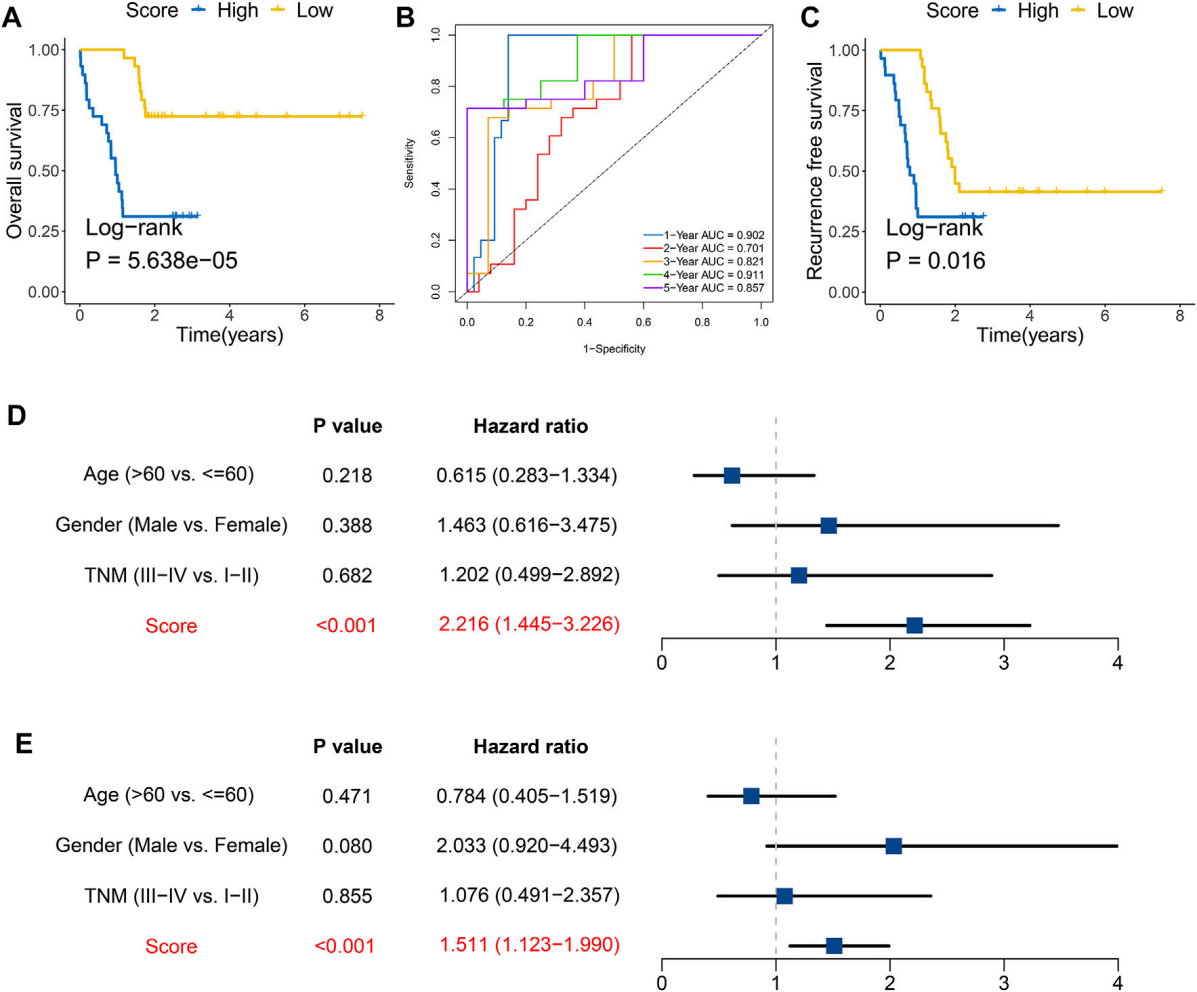
Validation of the FAIS *via* in-house cohort. **(A)** Kaplan–Meier curve of OS according to the FAIS in the in-house cohort. **(B)** Time-dependent ROC analysis for predicting OS at 1–5 years according to the FAIS. **(C)** Kaplan–Meier curve of RFS according to the FAIS in the in-house cohort. **(D,E)** Multivariate Cox regression analyses of OS **(D)** and RFS **(E)** in the in-house cohort.

To confirm the clinical implication of the FAIS, a clinical in-house cohort was used to evaluate its performance. Based on the median of risk score, patients were divided into high- and low-risk groups. As expected, patients in the low-risk group exhibited more favorable prognosis (Figure 5A). In the in-house cohort, the AUCs for predicting OS at 1–5 years were 0.902, 0.701, 0.821, 0.911, and 0.857 in FAIS, respectively (Figure 5B). The Kaplan–Meier analysis of RFS also indicated that the low-risk group had a favorable prognosis (Figure 5C). Multivariate Cox regression analyses verified that the FAIS was an independent predictive factor for OS and RFS (Figures 5D,E). All the results validated the excellent performance and reconfirmed the clinical applicability of FAIS.

Apart from the prognostic value, there were also differences in the distribution of clinicopathological characteristics and molecular alternations in the two groups. Compared to the low-risk group, most FAGs had higher expression in the high-risk group, whereas *FCN3* and *GADD45B* exhibited the opposite pattern (Supplementary Figure S3A). High expression of *FCN3* and *GADD45B* corresponded to a favorable prognosis, which was consistent with the above and previous results (Zhao et al., 2018; Wang et al., 2021). For clinicopathological characteristics, there were no conspicuously different distributions of age, gender, and BMI (Supplementary Figure S3B). The high-risk group mainly presented in patients with an advanced AJCC stage, a superior histological grade, and vascular invasion, predominantly related to the malignant phenotype and adverse prognosis (Supplementary Figure S3B). With the advancement of the AJCC stage and T stage, the FAIS score showed an increasing tendency, which presumed that the FAIS could stratify HCC patients, improving clinical outcomes (Supplementary Figures S3C,D). Overall, the FAIS could serve as an independent predictor of OS and RFS and help promote the prognostic management of HCC patients.

### The Landscape of Somatic Mutation and Copy Number Alteration in the Two Groups

**FIGURE 6:**
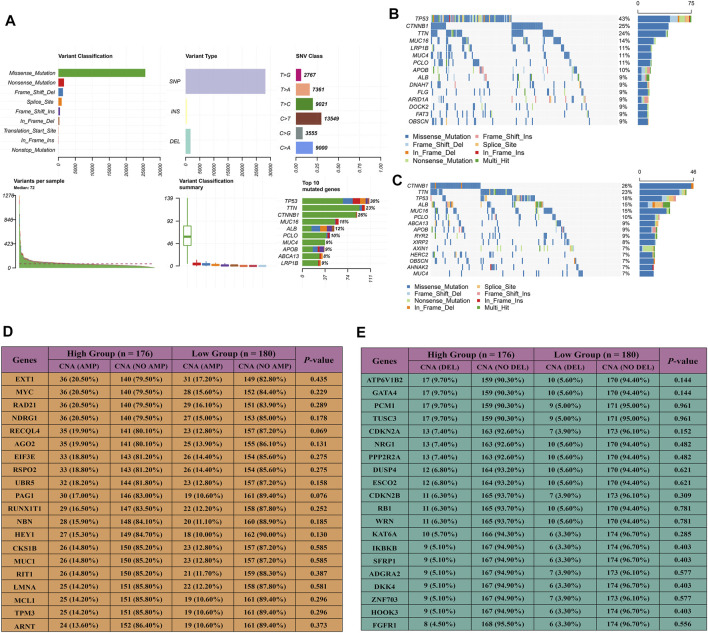
Genomic alterations of high-risk and low-risk groups in the TCGA cohort. **(A)**. Summary of somatic mutations for all HCC patients. **(B,C)** Top 15 significantly mutated genes in a high-risk group **(B)** and low-risk group **(C)**. The percentage on the right showed the proportion of samples with mutations. **(D,E)** Top 20 genes with significant amplification and deletion of copy number alteration (CNA). Between the high-risk group and low-risk groups, the difference of amplification **(D)** and deletion **(E)** rates are further compared. “Amp” means amplification of CNA. “DEL” means deletion of CNA.

We depicted and explored genomic alterations to identify distinct molecular characteristics in the two groups. A summary of somatic mutations for all HCC patients is exhibited in Figure 6A. The most universal features of variant classification, variant type, and single nucleotide variant (SNV) class were missense mutations, single-nucleotide polymorphisms (SNPs), and “C > T”. The variant numbers for each sample and the mutation frequency of the top 10 genes were rendered intuitively (Figure 6A). Using the maftools R package, the top 15 mutated genes were identified and defined as significantly mutated genes (SMGs) between the two groups (Figures 6B,C). Eight overlapping SMGs were present in the two groups, encompassing *TP53*, *CTNNB1*, *TTN*, *MUC16*, *MUC4*, *PCLO*, *ALB*, and *OBSCN*, implying that these alterations were prevalent in HCC. Notably, the tumor suppressor gene *TP53*, proto-oncogene *CTNNB1*, and the largest known protein-encoded gene *TTN* were the top three SMGs in both groups. The alterations of these genes might be important drivers of HCC progression. Except for the core gene of the cell cycle, *TP53*, there were no significant mutation differences of common SMGs between the two groups (*p-*value > 0.05), but the metabolic gene *ALB* showed a lower mutation frequency in the high-risk group relative to the low-risk group (Supplementary Figure S4). The mutation of *TP53* plays a key role in hepatocarcinogenesis and is associated with adverse clinical outcomes (Gao et al., 2019). The *ALB* gene is involved in both inflamed and metabolic pathway networks and impacts the prognosis of HCC (Chen et al., 2020; Wang et al., 2020). Thus, diverse mutations with specific molecules were associated with opposite clinical outcomes in the two groups. The amplification and deletion of copy number alteration (CNA) were further decoded. The top 20 genes were recruited based on copy number amplification and deletion for each patient. Both amplification and deletion were not pronounced (all *p*-value > 0.05), meaning that similar CNA events existed in the two groups (Figures 6D,E). These results suggested that somatic mutation might have more crucial impacts on distinct prognosis and HCC progression compared to CNA.

### Distinct Biological Functions in the Two Groups

**FIGURE 7:**
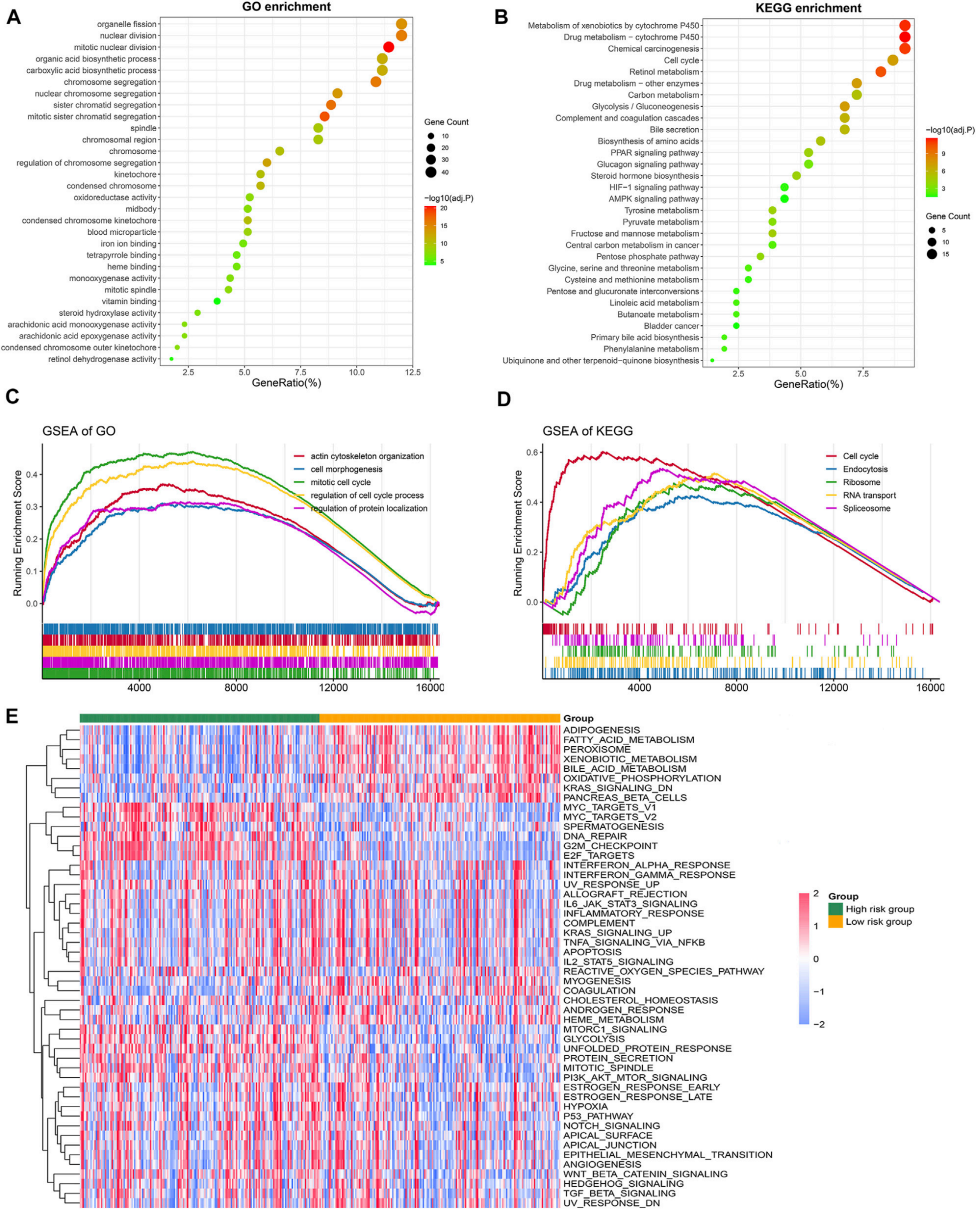
Distinct biological functions of the two groups. **(A,B)** GO **(A)** and KEGG **(B)** enrichment analysis of differentially expressed genes between the high-risk group and low-risk group. The top 30 significantly enriched pathways extracted with adjusted *p*-value < 0.05. **(C,D)** Enrichment plots depicted by gene set enrichment analysis (GSEA) based on GO **(C)** and KEGG **(D)** gene sets, respectively. **(E)** Heatmap of 50 Hallmark gene sets between the high-risk group and low-risk group using the GSVA algorithm.

The initiation and development of HCC are regulated *via* multiple biological pathways. Using the clusterProfiler and GSVA R package, we depicted the specific biological characteristics of the two groups. Based on DEGs, the GO enrichment analysis indicated that cell division and biosynthetic processes were conspicuously enriched (Figure 7A). The KEGG enrichment analysis suggested that cell cycle and metabolism-associated pathways were identified (Figure 7B). Due to the significant heterogeneity of HCC, the two groups might have various functional characteristics. Enrichment analysis (GSEA) was further conducted, and we discovered that the significantly enriched cell cycle pathway was a crucial feature of the high-risk group (Figures 7C,D). Consistent with previous studies, the cell cycle and metabolism were key biological functions in HCC (Wu et al., 2020). Moreover, we investigated the underlying carcinogenic characteristics of the two groups with the GSVA algorithm. The high-risk group was characterized as cell cycle-associated pathways, and the low-risk group was characterized as metabolism-associated functions (Figure 7E). These results indicated that the two groups displayed different biological characteristics and were influenced by multiple pathways.

### The Estimation of Immune Infiltration by the Fibrosis-Associated Signature

**FIGURE 8:**
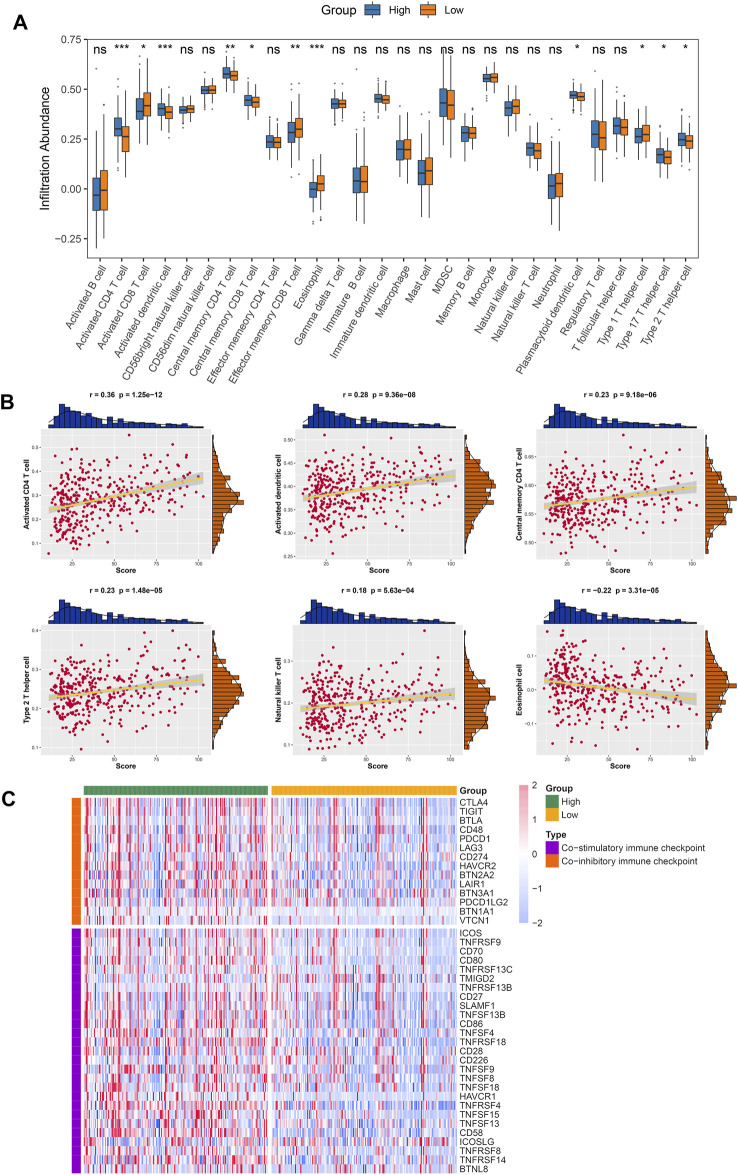
Landscape of immune cell infiltration and profiles of immune checkpoint. **(A)** Distribution of 28 immune cell infiltrations between two risk groups in the TCGA-LIHC cohort. **(B)** Correlations between six specific immune cells and risk score using Spearman analysis. **(C)** Expression heatmap of immune checkpoints between two risk groups in the TCGA-LIHC cohort. ns *p* >0.05; *p <0.05, **p < 0.01, ***p < 0.001.

Since inflammation and immune cells are key factors for the process of fibrosis (Mack, 2018) and the FAIS was established based on FAGs, we hypothesized that there were distinct immune characteristics in the two risk groups. The ssGSEA algorithm was utilized to assess the infiltration abundance of 28 immune cells. As instructed, the heatmap depicted the relative infiltration of different immune cell populations (Supplementary Figure S5A). Superior infiltration of active CD8^+^ T cells was the immune feature in the low-risk group, whereas pronounced higher levels of active CD4^+^ T cells and dendritic cells (DCs) were the immune characteristics of the high-risk group (Figure 8A). A previous study proved that CD8^+^ T cells, named cytotoxic T cells, were the most crucial factor in killing tumor cells (Maimela et al., 2019). Therefore, compared to the high-risk group, patients in the low-risk group had superior CD8^+^ cell infiltration and might benefit more from ICI treatment. In addition, we discovered that FAIS exhibited a broad positive association with different immune cell categories, except for eosinophil cells (Figure 8B and Supplemetary Figure S5B). The most significant correlation was found between FAIS and CD4^+^ T cells (Figure 8B).

### Immune Checkpoint Profiles of Fibrosis-Associated Signature and Prediction of Immunotherapy

**FIGURE 9:**
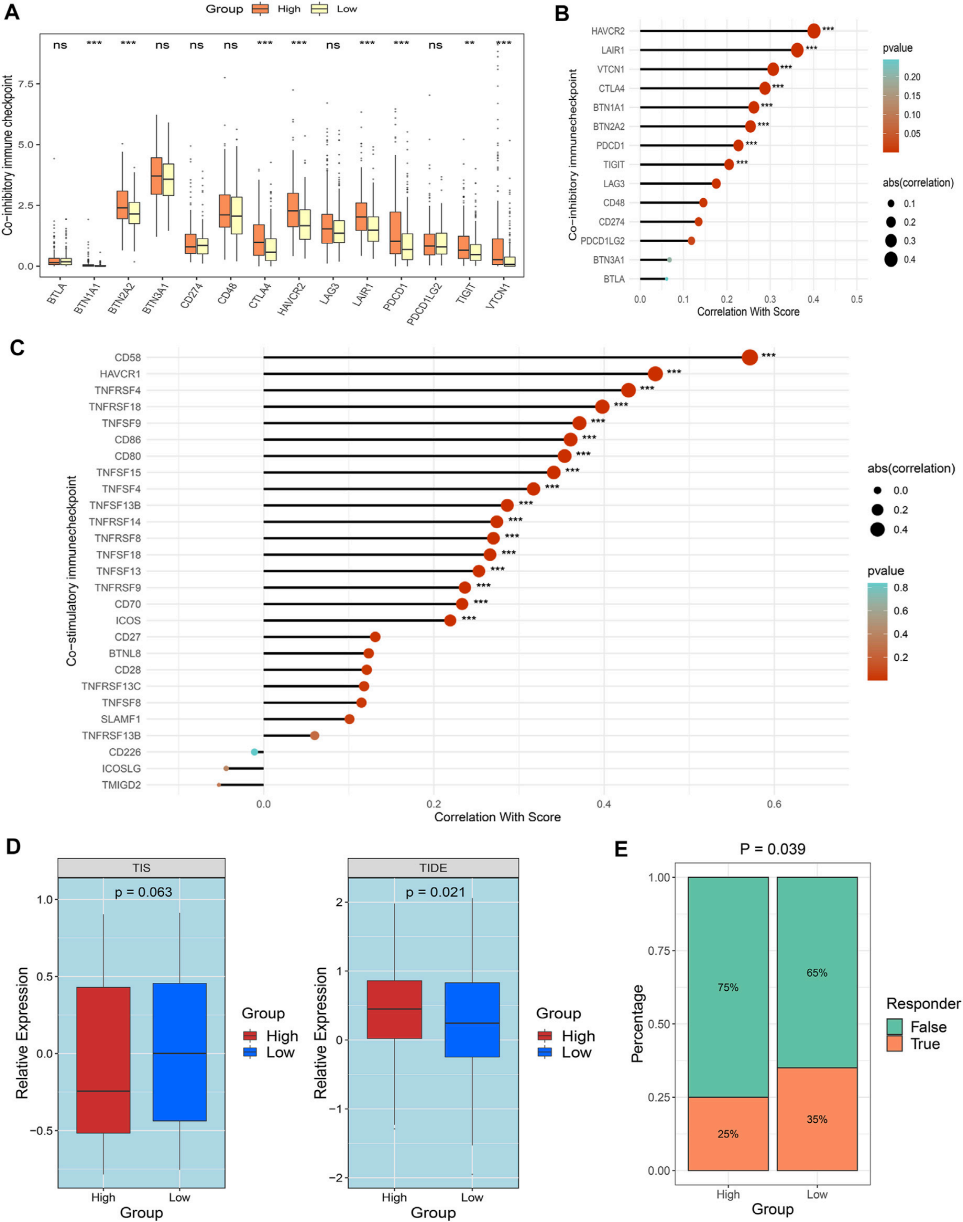
Deep exploration of immune checkpoints and potential immunotherapy predictor of FAIS. **(A)** Distribution of co-inhibitory ICPs between two risk groups in the TCGA-LIHC cohort. **(B)** Correlations between co-inhibitory ICPs and risk score using Spearman analysis. **(C)** Correlations between co-stimulatory ICPs and risk score using Spearman analysis. **(D)** Distribution difference of TIDE and TIS prediction scores between the high-risk group and low-risk group. **(E)** Immunotherapy response ratio of FAIS in the TCGA-LIHC cohort. ns *p* > 0.05; *p < 0.05, **p < 0.01, ^***^ *p* < 0.001.

The landscape of immune checkpoints in the two groups was decoded to gain new insights into facilitating clinical practice. Both co-stimulatory and co-inhibitory immune checkpoints (ICPs) exhibited higher expression in the high-risk group (Figure 8C). Prior studies suggested that the higher the expression of ICPs is the more immune suppression there is, further resulting in immune tolerance and unfavorable prognosis (Das and Johnson, 2019). Screening specific ICPs in the high-risk group might yield more benefit from ICI therapy. For most co-inhibitory ICPs, patients in the high-risk group displayed superior expression, such as *CTLA4* and *TIGIT* (Figure 9A). Moreover, the risk score was broad and positively correlated with the expression of co-inhibitory ICPs (Figure 9B). Consistent with this finding, patients in the high-risk group also exhibited higher expression of most co-inhibitory ICPs (Supplementary Figure S5C). A strongly positive correlation was also found between the risk score and most co-inhibitory ICPs (Figure 9C). Notably, the tumor necrosis factor receptor superfamily (TNFRSF) showed conspicuously higher expression in the high-risk group. Targeting these specific molecules might have stronger potential benefits for patients in the high-risk group. To further evaluate the immunotherapeutic efficacy of the two groups, we applied TIS and TIDE approaches. The results indicated that a higher TIS score and lower TIDE score were characteristics of the low-risk group, implying that these patients might obtain more considerable benefits and improved clinical outcomes (Figure 9D). The patients in the low-risk group had a higher proportion of responders to immunotherapy (Figure 9E). Overall, the patients in the low-risk group demonstrated a better immunotherapy response.

## Discussion

Hepatocellular carcinoma (HCC) is a complex disease with elevated incidence and mortality (Villanueva, 2019; Craig et al., 2020). A previous study reported that there are three distinct TME phenotypes in HCC that are prominently associated with prognosis, immune surveillance, immune escape, and genetic alterations (Liu et al., 2021e). Liver fibrosis indicates tremendous potential in tumorigenesis and tightly correlates with prognosis (Kariyama et al., 2019). Inflammation bursts and the immune responses activate hepatic stellate cells (HSCs) and favor extracellular matrix (ECM) deposition, leading to liver fibrosis (Koyama and Brenner, 2017; Mack, 2018). For instance, Th17 cells produce IL-17 cytokines and implicate fibrogenesis, and dendritic cells (DCs) induce HSCs to mediate inflammation and fibrogenesis (Koyama and Brenner, 2017). In parallel, immune cells are a major component of TME that have profound impacts on prognosis, carcinogenesis, and immunotherapy (Liu et al., 2021e). Therefore, liver fibrosis might interfere with TME phenotypes and the shape of HCC heterogeneity and even has implications for the immunotherapy response. Revolutionized and thrilling progress has been achieved in immunotherapy, but prominent therapeutic efficacy is limited to a small subset of populations. It is imperative to increase the understanding of HCC heterogeneity and search for a stable tool for identifying patients with excellent immunotherapy response.

Using three machine learning algorithms, a robust FAIS was ultimately constructed based on FAGs. The advantage of integrative analysis and comparison by multiple approaches arises in making the model more applicable and translational. This study shows that the FAIS was an independent factor for OS and RFS, which could serve as a prognostic biomarker. According to previous research, most of the 11 genes included in the FAIS displayed pivotal roles in the prognosis and progression of HCC (Liu et al., 2016a; Qu et al., 2017; Wang et al., 2021). *FCN3* was reported to mediate apoptosis, activate the complement lectin pathway, regulate the immune system, and be a potential immunotherapy target and prognosticator for HCC (Wang et al., 2021). *SPP1* was highly expressed in HCC and contributed to tumorigenesis by promoting a stem-like phenotype (Liu et al., 2016a). *MCM7* regulated the initiation of DNA replication and driven HCC progression by cyclin D1-dependent signaling (Qu et al., 2017). There was strong attraction to further decipher the characteristics of HCC patients, and we comprehensively explored genomic alterations, clinical features, biological functions, and the immune landscape.

Patients were assigned into two groups by FAIS, the high-risk group with the features of an advanced AJCC stage, superior histological grade, and vascular invasion and the low-risk group with the traits of an early AJCC stage, inferior histological grade, and vascular invasion. Mostly, patients in the high-risk group were linked with poor prognosis, thus facilitating clinical management. Furthermore, metabolic disorders and aberrant proliferation are ubiquitous in liver diseases (Piccinin et al., 2019). The underlying biological pathways of the two groups are depicted in detail. The high-risk group was mainly enriched in metabolism-associated pathways, and the low-risk group was significantly enriched in proliferation-related pathways. Notably, patients in the high-risk group with adverse prognosis possessed the highest somatic mutation frequency, 43%, for the TP53 mutation. The DNA-replication kinase CDC7 inhibitor selectively treated liver cancer cells with TP53 mutation, inducing cell senescence (Wang et al., 2019). Molecular targeted therapies are increasingly prevalent in cancer treatment (Klinke, 2010). Distinct molecular alterations could be used as potential therapeutic targets, particularly for patients with poor prognosis and more molecular variations.

As an emerging and potential therapeutic approach, immunotherapy is gradually being incorporated into the treatment armamentarium in HCC (Llovet et al., 2018). Currently, because immunotherapy response lacks effective assessments, a promising indicator is urgently needed to improve the predictive performance of immunotherapy in HCC. The immune landscape and immune checkpoint profiles were dissected to identify the populations that may benefit. Our results suggested that patients in the low-risk group might yield more benefit from immunotherapy due to superior CD8^+^ T cell infiltration. ICI therapy is the backbone of immunotherapy aimed at enhancing the durable response and survival of patients with HCC and employs monoclonal antibodies targeting immune checkpoints, such as *PD1* and *PD-L1* (Cheng et al., 2020). Using TIDE and TIS, we found that patients in the low-risk group had a better immunotherapy response. Co-stimulatory and co-inhibitory ICPs are critical immune molecules that participate in T cell activation. The co-inhibitory ICPs attenuated anti-tumoral immune responses and facilitated tumor immune escape by suppressing T cell functions (Sangro et al., 2020). In the high-risk group, the expression of co-inhibitory ICPs was more highly and widely positively associated with FAIS. The aberrant distribution of co-inhibitory ICPs not only supported that these patients had worse prognosis but also implied that targeting these specific molecules *via* ICI treatment may improve clinical outcomes. Notably, the co-stimulatory ICPs were significantly elevated in the low-risk group and exhibited an extensive positive correlation with FAIS, especially for TNF superfamily members. *TNFRSF9* (also known as *4-1BB*) is conducive to T cell activation and augments tumor immunity. The agonist anti-*4-1BB* mAbs demonstrates the capacity to enhance CD8^+^ T cell responses to promote tumor rejection (Buchan et al., 2018; Pourakbari et al., 2021). *TNFRSF18* (also known as *GITR*) combines with the *GITR* ligand to favor T cell proliferation, and an anti-*GITR* antibody agonist (TRX518) displays potent anti-tumor activity (Zappasodi et al., 2019). In addition, with the development of co-stimulation agonist therapy, combination therapy with ICIs is a booming immunotherapy approach. For patients in the high-risk group, co-stimulation agonists would be a sensible option. These results indicated that FAIS could stratify patients and guide precise treatment to some degree as a promising biomarker.

Although FAIS displayed many strengths, there are also some limitations. First, all cohorts retrieved in this study were retrospective, and further validation of this risk scoring system should be applied to prospective cohorts. Second, our study used bioinformatics algorithms to indirectly evaluate the immunotherapy response rather than large-scale immunotherapy clinical trials. Third, patients in the high-risk group had inferior prognosis and poor immunotherapy response, which needed more attention. In the further research, we will focus on these populations to develop more effective therapy strategies.

## Conclusion

From the perspective of fibrosis, an independent predictor of OS and RFS, the FAIS was constructed by three machine learning algorithms. The FAIS could stratify HCC patients who had distinct genomic alterations, clinical features, biological functions, and immune landscapes. Our findings also revealed that FAIS had critical implications for immunotherapy and might be a useful tool to favor clinical surveillance and management for individual patients.

## Data Availability

The original contributions presented in the study are included in the article/Supplementary Material, and further inquiries can be directed to the corresponding author.

## Supplementary Material

Supplementary Table S2 The gene sets for marking 28 immune cell types.

Supplementary Table S3 The forward and reverse primers for quantitative real-time PCR.
