# Supplementary material for: An Integrated Fibrosis Signature for Predicting Survival and Immunotherapy Efficacy of Patients With Hepatocellular Carcinoma
Source: Front Mol Biosci. 2021 Dec 14;8:766609. doi: 10.3389/fmolb.2021.766609 (PMC8712696; doi:10.3389/fmolb.2021.766609)
Supplement: Supplementary file 2 [file DataSheet2.docx]

**Supplementary Method**

**Quantitative Real-Time PCR (qRT-PCR)**

Using RNAiso Plus reagent (Takara, Dalian, China), total RNA was obtained and isolated from HCC tissues. The RNA quality was assessed by a NanoDrop One C (Waltham, MA, USA). According to the manufacturer's instructions, an aliquot of 1 μg of total RNA was reverse transcribed into complementary DNA (cDNA) using a High-Capacity Cdna Reverse Transcription Kit (TaKaRa Bio, Japan). Quantitative real-time PCR (qRT-PCR) was performed using SYBR Assay I Low ROX (Eurogentec, USA) and SYBR® Green PCR Master Mix (Yeason, Shanghai, China) to detect gene expression. The qRT-PCR assays were performed in triplicate with the following conditions: (1) 95°C for 5 min and (2) 40 cycles of 95°C for 10 s and 60°C for 30 s. The expression value was normalized to *GAPDH* and calculated using the ΔCT (Ct mRNA-Ct *GAPDH*) method, and then log2 transformed for subsequent analysis. The sequences of qRT-PCR primers were shown in Table S3.
