# Supplementary material for: An Integrated Fibrosis Signature for Predicting Survival and Immunotherapy Efficacy of Patients With Hepatocellular Carcinoma
Source: Front Mol Biosci. 2021 Dec 14;8:766609. doi: 10.3389/fmolb.2021.766609 (PMC8712696; doi:10.3389/fmolb.2021.766609)
Supplement: Supplementary file 3 [file DataSheet1.docx]

**Supplementary Figures**

- Figure S1
- Figure S2
- Figure S3
- Figure S4
- Figure S5


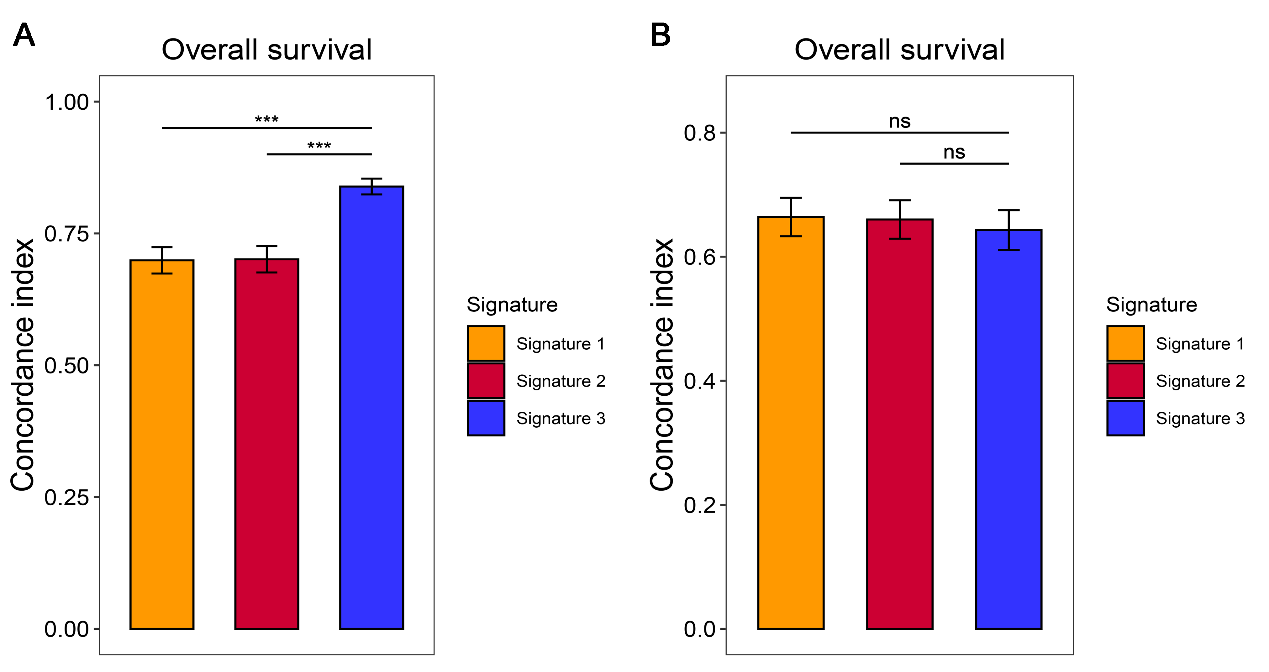


**Figure S1. The Harrell’s C-index of signatures in TCGA-LIHC and GSE14520 cohorts.** C-index of signature-1, signature-2, and signature-3 for evaluating OS in TCGA-LIHC **(A)** and GSE14520 cohort **(B)**. *^ns^ P >0.05;* ^*^*P* <0.05, ^**^P <0.01, ^***^P <0.001.


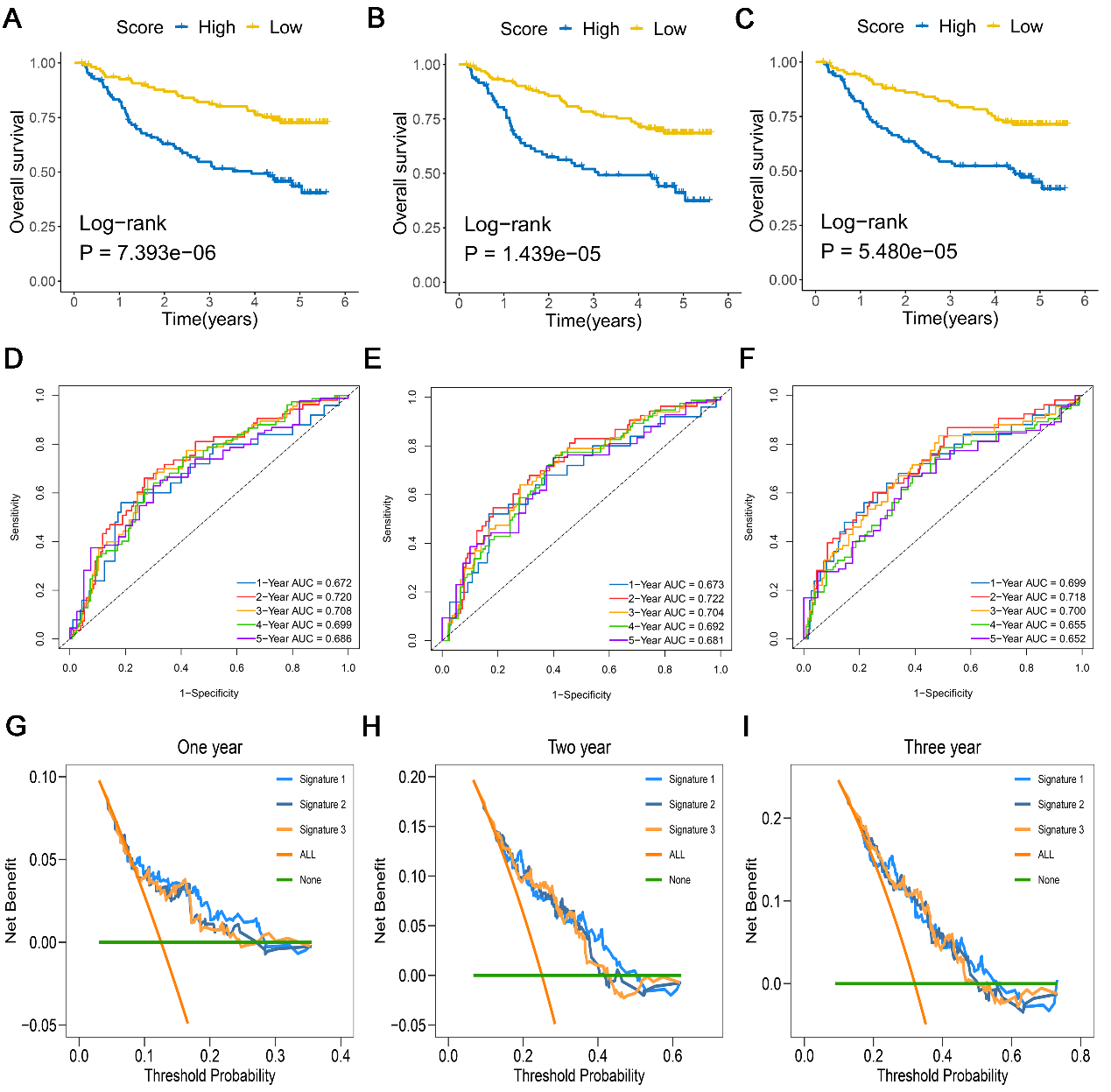


**Figure S2.** **Validation and comparison of signatures in GSE14520 cohort. A-C.** Kaplan-Meier curves of OS according to the signature-1 **(A)**, signature-2 **(B)**, and signature-3 **(C)**. **D-F.** Time-dependent ROC analysis for predicting OS at 1~5 years according to the signature-1 **(D)**, signature-2 **(E)**, and signature-3 **(F)**. **G-I.** DCA curves of signatures for evaluating 1- **(G)**, 2- **(H)**, and 3-year **(I)** OS, respectively.


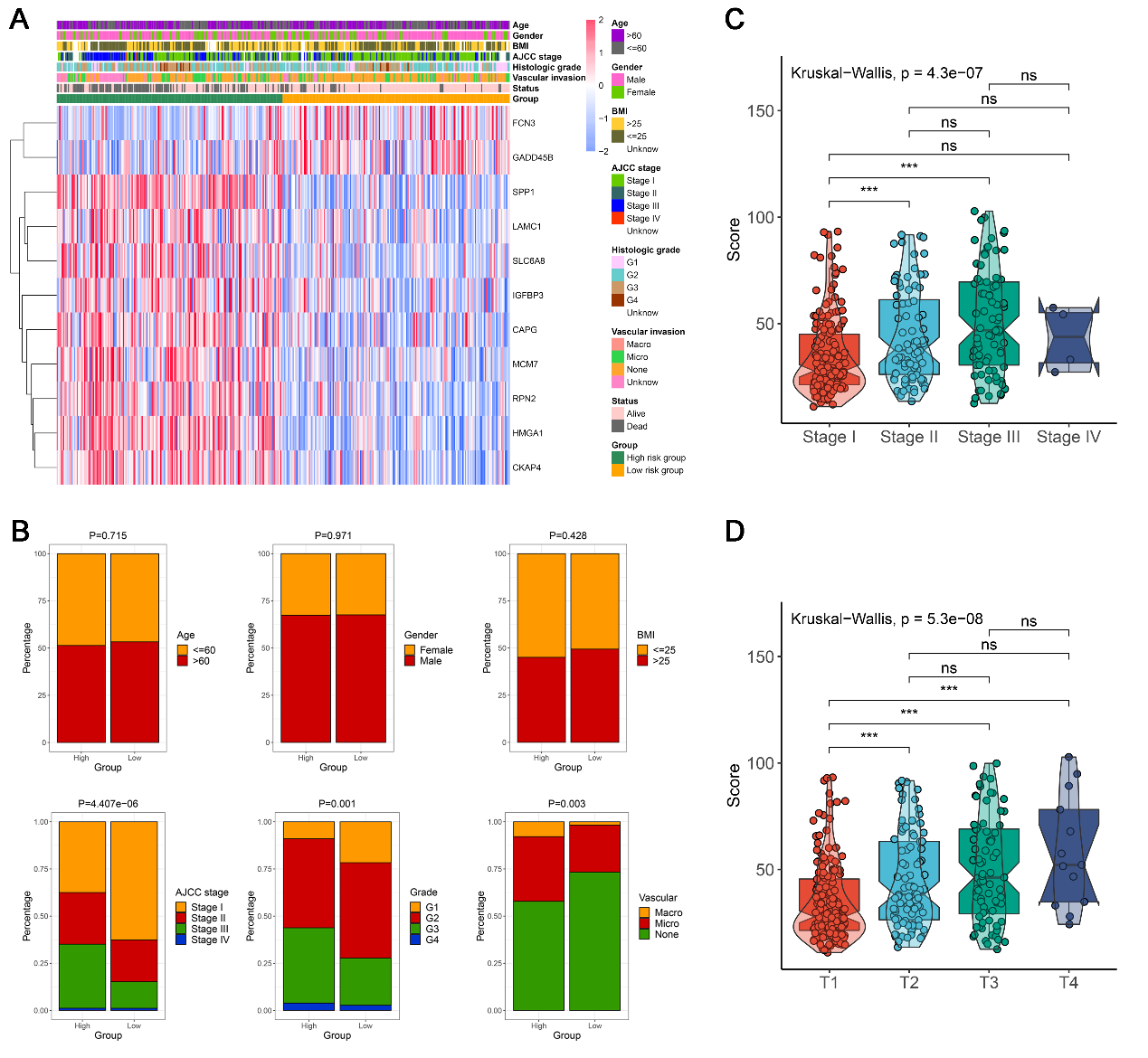


**Figure S3.** **The clinical characteristics of FAIS in TCGA-LIHC cohort. A.** The expression heatmap of 11 FAGs enrolled in signature. Survival status, age, gender, BMI, AJCC stage, histology grade, and vascular invasion were displayed as patient annotations. **B.** Composition percentage of clinical characteristics, including age, gender, BMI, AJCC stage, histology grade, and vascular invasion. **C-D.** The distribution of risk score among distinct AJCC stages **(C)** and T stages **(D)**. *^ns^ P >0.05;* ^*^*P* <0.05, ^**^P <0.01, ^***^P <0.001.


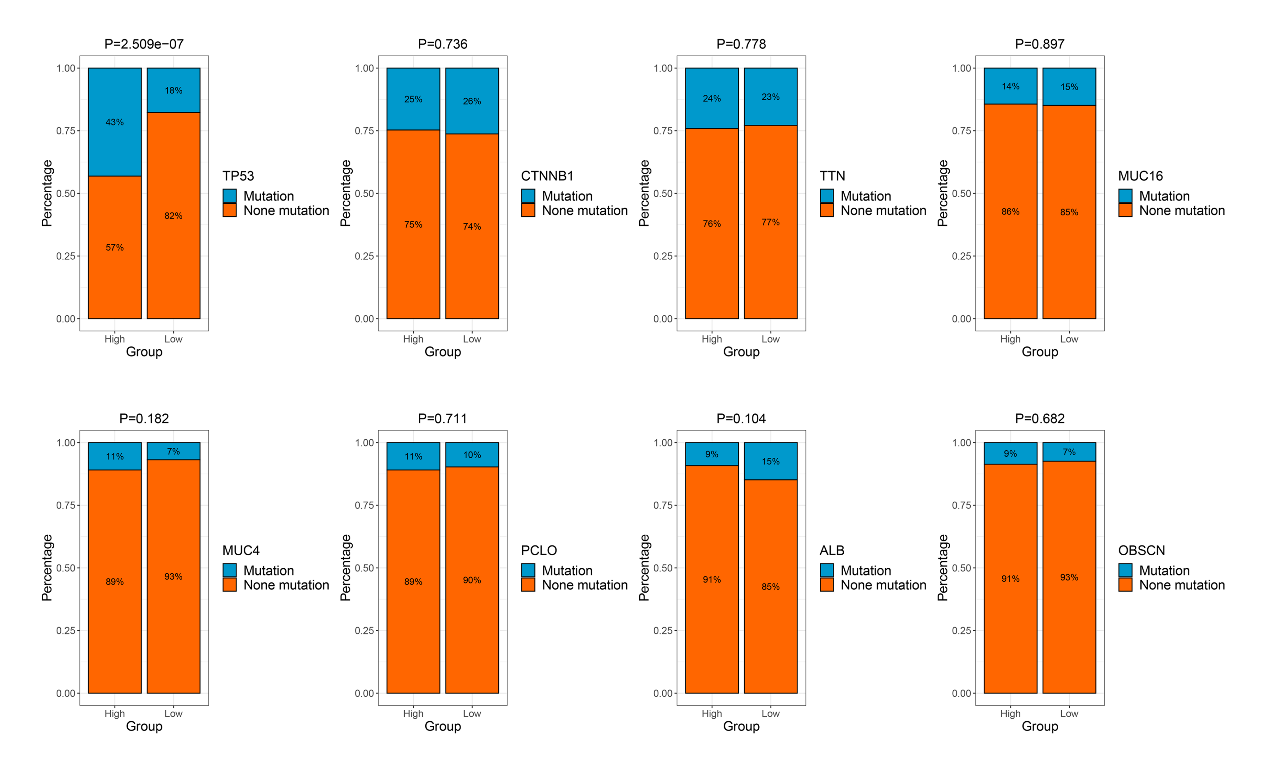


**Figure S4. The molecular** **characteristics of FAIS in TCGA-LIHC cohort.**


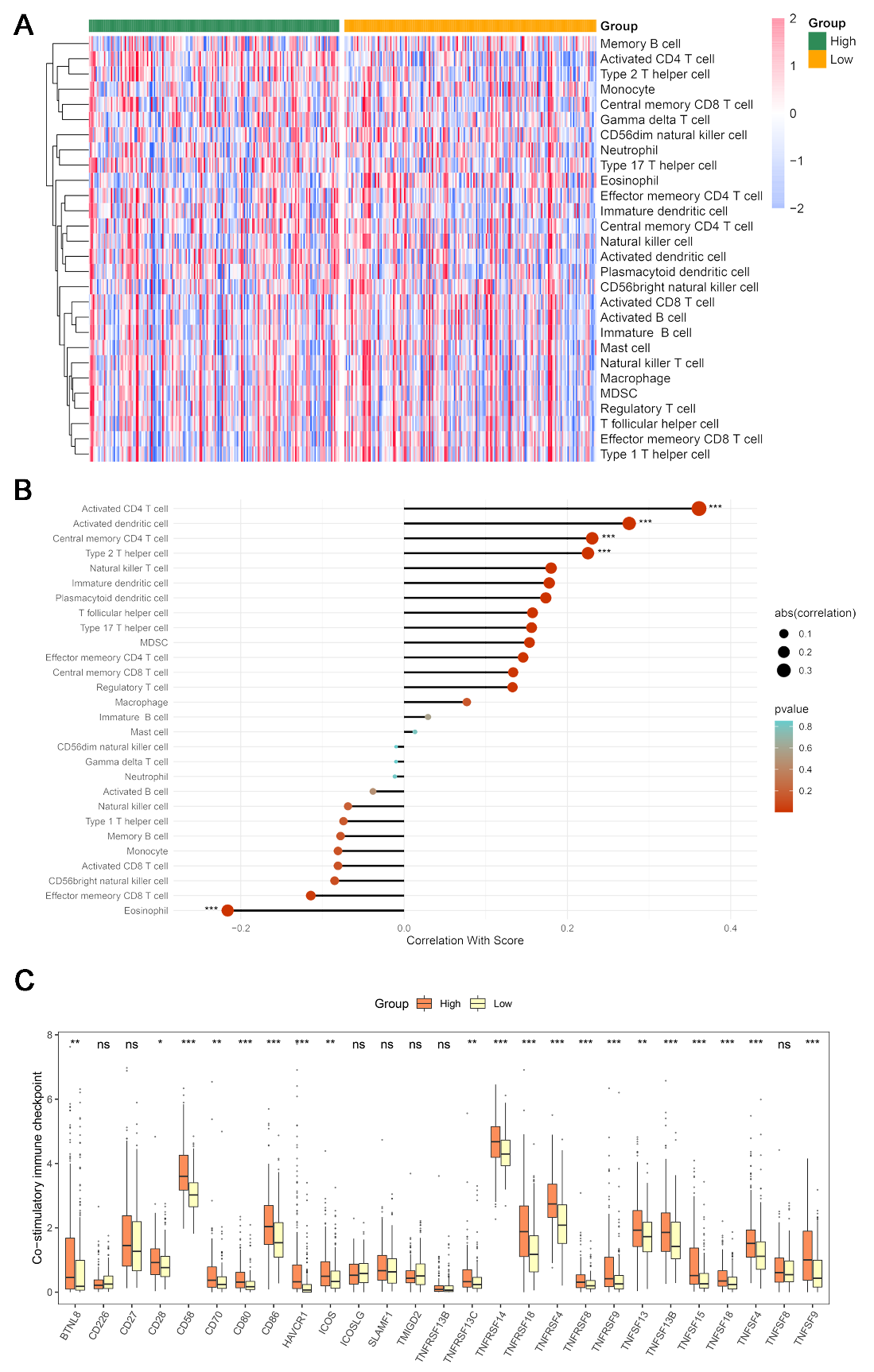


**Figure S5. The distinct features of immune cells infiltration and co-stimulatory ICPs expression. A.** The infiltration abundance of 28 immune cell subsets evaluated by ssGSEA algorithm for two groups. **B.** Correlations between immune cell infiltration and risk score using Spearman analysis. **C.** The distribution difference of co-stimulatory ICPs between the high-risk and low-risk groups. *^ns^ P >0.05;* ^*^*P* <0.05, ^**^P <0.01, ^***^P <0.001.
